# The Role of Machine Learning in Diagnosing Bipolar Disorder: Scoping Review

**DOI:** 10.2196/29749

**Published:** 2021-11-19

**Authors:** Zainab Jan, Noor AI-Ansari, Osama Mousa, Alaa Abd-alrazaq, Arfan Ahmed, Tanvir Alam, Mowafa Househ

**Affiliations:** 1 College of Health and Life Sciences Hamad Bin Khalifa University Qatar Foundation Education City, Doha Qatar; 2 Division of Information and Computing Technology, College of Science and Engineering Hamad Bin Khalifa University Qatar Foundation, Education City Doha Qatar; 3 Department of Psychiatry, Weill Cornell Medicine Education City, Doha Qatar

**Keywords:** machine learning, bipolar disorder, diagnosis, support vector machine, clinical data, mental health, scoping review

## Abstract

**Background:**

Bipolar disorder (BD) is the 10th most common cause of frailty in young individuals and has triggered morbidity and mortality worldwide. Patients with BD have a life expectancy 9 to 17 years lower than that of normal people. BD is a predominant mental disorder, but it can be misdiagnosed as depressive disorder, which leads to difficulties in treating affected patients. Approximately 60% of patients with BD are treated for depression. However, machine learning provides advanced skills and techniques for better diagnosis of BD.

**Objective:**

This review aims to explore the machine learning algorithms used for the detection and diagnosis of bipolar disorder and its subtypes.

**Methods:**

The study protocol adopted the PRISMA-ScR (Preferred Reporting Items for Systematic Reviews and Meta-Analyses Extension for Scoping Reviews) guidelines. We explored 3 databases, namely Google Scholar, ScienceDirect, and PubMed. To enhance the search, we performed backward screening of all the references of the included studies. Based on the predefined selection criteria, 2 levels of screening were performed: title and abstract review, and full review of the articles that met the inclusion criteria. Data extraction was performed independently by all investigators. To synthesize the extracted data, a narrative synthesis approach was followed.

**Results:**

We retrieved 573 potential articles were from the 3 databases. After preprocessing and screening, only 33 articles that met our inclusion criteria were identified. The most commonly used data belonged to the clinical category (19, 58%). We identified different machine learning models used in the selected studies, including classification models (18, 55%), regression models (5, 16%), model-based clustering methods (2, 6%), natural language processing (1, 3%), clustering algorithms (1, 3%), and deep learning–based models (3, 9%). Magnetic resonance imaging data were most commonly used for classifying bipolar patients compared to other groups (11, 34%), whereas microarray expression data sets and genomic data were the least commonly used. The maximum ratio of accuracy was 98%, whereas the minimum accuracy range was 64%.

**Conclusions:**

This scoping review provides an overview of recent studies based on machine learning models used to diagnose patients with BD regardless of their demographics or if they were compared to patients with psychiatric diagnoses. Further research can be conducted to provide clinical decision support in the health industry.

## Introduction

### Background

Bipolar disorder (BD) is a predominant mental disorder that involves dramatic shifts in mood and temper. It is the 10th most common cause of frailty in young adults and affects approximately 1% to 5% of the overall population [1]. It is mostly initiated during emotional states caused by disturbances in thinking, ranging from extreme mania and excitement to severe depression [2]. An epidemiological survey reported that its prevalence is rapidly increasing every year [3]. BD is associated with an evidently higher early mortality [4]. Bipolar patients have unfortunate life situations because these patients have a lifetime 9 to 17 years lower than that of normal people [5]. Additionally, several studies from various countries including Denmark and the United Kingdom state that this mortality difference has continuously been increasing since the last decades [6]. Although the maximum number of death cases in BD are due to cardiovascular diseases and diabetes, some death cases are due to unnatural events. Suicide is also relatively predominant in the patients with BD [6]. Suicide rates in patients with BD are 10%-20% higher than in the general population [4]. This context demonstrates significant background knowledge on bipolar disorder.

To effectively comprehend BD conditions and stipulate better treatment, primary exposure to mental disorders is a crucial phase. Different from finding other long-lasting situations that depend on laboratory trials and statistical analysis, BD is stereotypically detected based on patients’ self-statements in precise surveys planned for uncovering specific types of feelings, moods, and public relations [4]. Owing to the growing accessibility of information relating to patients’ mental health levels, artificial intelligence (AI) and machine learning (ML) skills are proving useful for deepening our comprehension of mental health situations, and they are promising methods to support psychiatrists in making better clinical decisions and analyses [7]. In recent years, AI techniques have shown superior performance in countless data-rich implementation frameworks, including BP [8,9].

In a previous review, Diego et al [10] discussed the applications of ML algorithms in diagnosing BD. They focused on 5 main application domains of ML in BD: diagnosis, prognosis, treatment, data-driven phenotypes plus research, and clinical direction. In contrast, the current review aims to evaluate existing literature on the applications of ML in BD diagnosis. Moreover, in the current review, we only focused on the role of ML in diagnosing BD and its types, which has not been previously comprehensively reviewed in any other study. We also discuss the strengths and challenges associated with the present work, future research guidelines for spanning the breach among the applications of ML procedures and patient diagnosis.

### Research Problem

BD is misdiagnosed as depressive disorder that leads to difficulties and delay in the treatment of affected patients [1]. Approximately 60% of patients with BD are looking for treatment of major depressive disorders [11]. According to a National Chinese Mental Health Survey report, while the incidence of BD in China increased by 4.5% within a 12-month period, the recognition rate of BD as a depressive disorder increased to 39.9% [12]. Hence, there is an urgent demand to diagnose BD correctly. Moreover, ML increasingly provides various advanced methods to diagnose BD at the individual level to achieve better clinical results [10]. Many scientists have used support vector machine (SVM) algorithms to build BD classification models using neuroimaging information to differentiate BD from major depression [13]. In Taiwan, scientists have designed prediction algorithms using random forests that calculate the genetic risk scores of BD [14]. However, based on all the evidence, it is necessary to provide a scoping review that focuses on all applications of ML for BD diagnosis. The current review aims to explore how ML algorithms are used for better diagnosis of BD.

## Methods

### Review Approach

The current scoping review was conducted to provide an understanding regarding the role of ML in diagnosing BD. A scoping review is an approach that is systematically executed to enable researchers to examine emerging evidence from available studies on a specific topic [15]. It is also helpful for identifying knowledge gaps in a given field [15]. This scoping review follows the PRISMA-ScR (Preferred Reporting Items for Systematic Reviews and Meta-Analyses Extension for Scoping Reviews) guidelines recommended in 2016 [16].

### Search Strategy

#### Search Sources

We conducted a systematic search in 3 electronic databases: PubMed, Google Scholar, and ScienceDirect. We searched for articles published between January 2016 and December 2021. The search was conducted between March 16 to March 20, 2021. The references lists of the included articles were reviewed to check for possible articles that could be included.

#### Search Terms

The search strategies applied differed depending on the nature of the databases chosen for the search and are given in Multimedia Appendix 1. For example, PubMed allows the application of limiters such as “humans” and “English” language articles. In addition, further search terms for BD were added as we uploaded the references of Medical Subject Headings (MeSH) in PubMed. Google Scholar and ScienceDirect limit the number of search terms. Therefore, some search terms were not used when searching in these 2 databases. The intervention terms identified were (“Artificial Intelligence*” OR “Deep Learning” OR “Machine Learning” OR “Natural Language Processing” OR neural network* OR “unsupervised learning” OR “supervised learning”). The disorder terms identified were (“Bipolar disorder” OR “Bipolar 1 Disorder” OR “Bipolar 2 Disorder” OR “bipolar mood disorder” OR “bipolar affective disorder” OR “Cyclothymic Disorder” OR Cyclothym* OR manic*. Regarding search terms related to studies’ outcome, which was bipolar disorder diagnosis, the search terms used were (diagnos* OR recog* OR prognosis OR detect* OR screening*).

The articles obtained from the search were uploaded to the Rayyan intelligent review application (Rayyan Systems Inc) in an EndNote (Clarivate) format [17]. This application allows researchers to collaborate and review articles at easily and at a faster pace [17]. Reviewers can create individual or collaborative reviews and make decisions regarding including or excluding the articles independently [17]. We considered 2 aspects when determining the key terms to be used for the current scoping review, which were population and interventions. The population we considered comprised Individuals with or without any health condition regardless of their gender, age, and ethnicity. The interventions considered include the ML models and algorithms used for diagnosing BD. The search terms were selected based on several scoping and systematic reviews we encountered during the preliminary search phase in the databases specified above.

#### Study Eligibility Criteria

Articles met the inclusion criteria if they achieved the main objective, namely providing an overview on the role of ML in diagnosing BD. The criteria identified for the inclusion and exclusion phases are given in Textbox 1.

**Criteria for study selection.**

**Inclusion criteria**

- Empirical studies
- Peer-reviewed articles, theses, dissertations, and reports
- No restrictions related to machine learning algorithms and models
- No restrictions on country of study
- English language
- No restrictions related to population
- Bipolar disorder

**Exclusion criteria**

- Newspapers, magazines, reviews, proposals, and posters
- Any language other than English
- Machine learning algorithms that do not detect bipolar disorder
- Nonhuman subjects

### Study Selection

In the first phase, 3 researchers (NA, OM, and ZJ) screened the titles and abstracts of the retrieved articles in an independent manner. In the second phase, the reviewers went through the full text of the articles included from the first phase. The retrieved articles were uploaded to the Rayyan intelligent review application in an EndNote format [17]. Disagreements were discussed amongst the 3 reviewers and decisions were made via consensus.

### Data Extraction

For data extraction, a form was developed to include all the different data considered for the scoping review such as the ML model, accuracy, and type of data used. A description of the data extraction fields is included in Multimedia Appendices 2 and 3. Data extraction was performed independently by the 3 reviewers (NA, OM, and ZJ) using and Microsoft Excel (Microsoft Corporation). Any disagreements regarding the extracted data were resolved via consensus. A summary of all the data extracted from included studies is given in Multimedia Appendices 4.

### Data Synthesis

This scoping review follows a narrative synthesis approach to synthesize the extracted data of the studies that made it to the final phase of inclusion and exclusion. From this analysis, we included studies that used ML models to assess participants with BD compared with other psychiatric disorders and healthy controls. The studies were classified based on the ML model used to diagnose BD, whether the model was an existing one or a novel one, BD type, data used, accuracy of diagnosis, other statistical measures, and whether the data used were private (gathered by the researchers) or public (open-access data). We also summarized the characteristics of the selected articles. Furthermore, we categorized the ML models into 10 categories and identified the characteristics of the selected studies that fitted under each category for the diagnosis of BD.

## Results

### Search Outcomes

**Figure 1:**
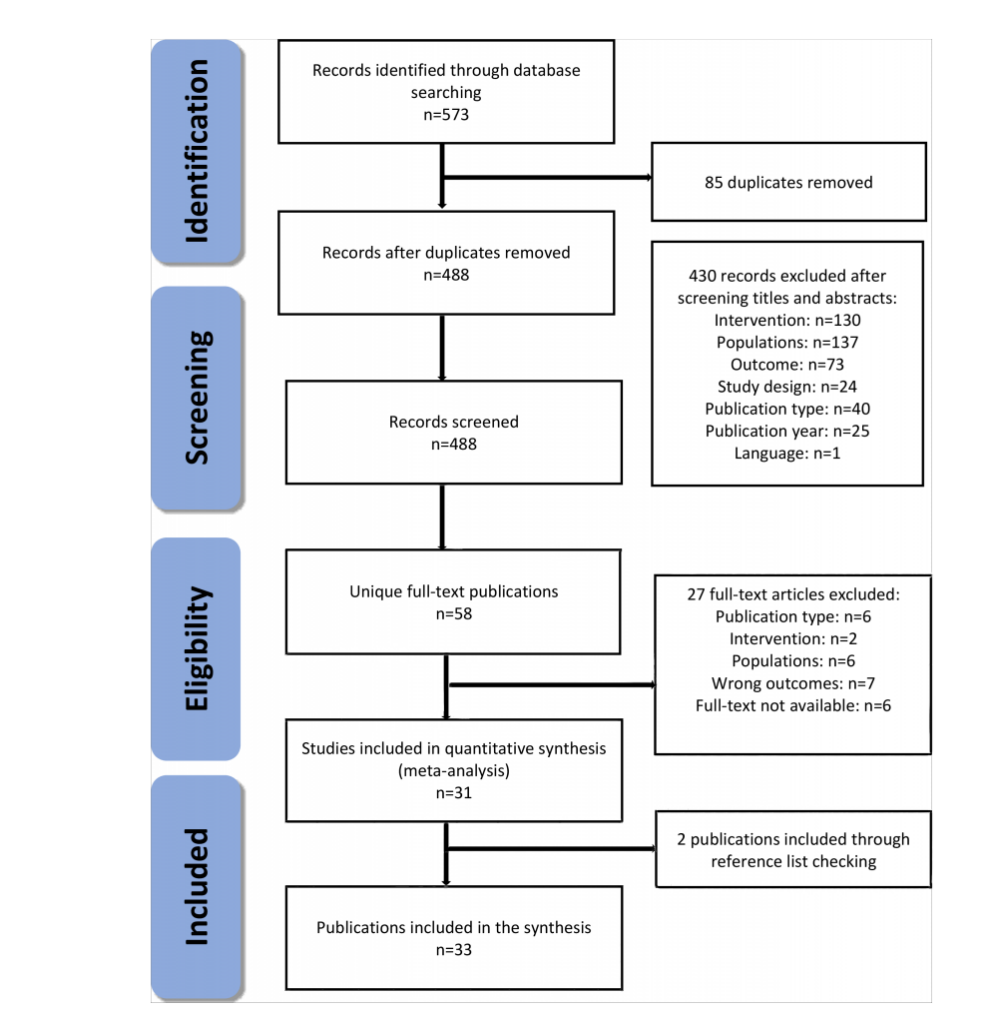
PRISMA (Preferred Reporting Items for Systematics Reviews and Meta-Analyses) flow diagram.

In this scoping review, we retrieved 573 potential articles from 3 different databases and included 33 studies for data synthesis, as shown in Figure 1. Among these, 488 articles remained after eliminating 85 duplicates. In the first phase of screening the titles and abstracts of the articles, 430 records (wrong intervention=130 articles, population=137 articles, outcome=73 articles, study design=24 articles, publication types=40 articles, publication year=25 articles, and language=1 article) were excluded. In the second phase, we reviewed the full text of 58 articles and included 31 articles. Then, 2 additional studies were added after checking the reference lists. Finally, 33 articles were selected for data synthesis.

### Characteristics of the Included Studies

**Figure 2:**
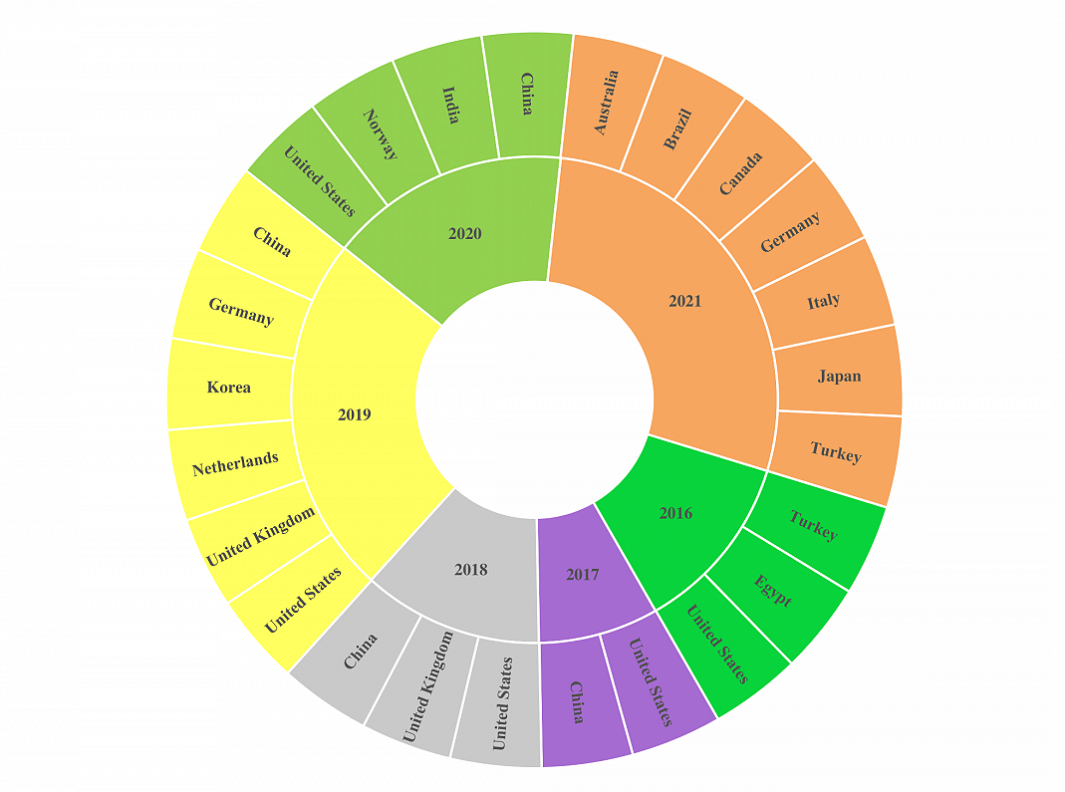
Publications by year and country.

Among the 33 included articles, 30 were research articles (91%) [14,18-46], whereas 3 articles were conference proceedings (9%) [24,42,47], as shown in Table 1 and Multimedia Appendix 4. Articles were published in 14 different countries; China (8, 24%) [14,18-20,22,25,30,39], India (1, 3%) [21], Germany (2, 6%) [23,47], United Kingdom (1, 3%) [26], United States (8, 24%) [27,28,32,34,37,38,41,45], Korea (2, 6%) [29,36], Egypt (1, 3%) [31], Turkey (2, 6%) [31,43], Italy (1, 3%) [33], Brazil (1%) [47], Australia (1%) [35], the Netherlands (1, 3%) [36], Norway (1, 3%) [37], Canada (1, 3%) [40] and Japan (1, 3%) [46]; however, the highest numbers of articles were from China and the United States, as observed in Figure 2. The highest numbers of the articles were published in 2018 and 2019 (7, 21.21%), as shown in Figure 2 and Multimedia Appendices 5 and 6. The basic purposes of the included studies were model development (24, 73%), evaluation (5, 16%), data analysis (3, 9%), and model adaptation (2, 6%) for the diagnosis of BD. In the included studies, different types of BD were diagnosed using ML techniques such as BD type 1 (27, 81%), BD type 2 (27, 82%), psychotic bipolar (3, 9%), chronic bipolar (2, 6%), and first episode bipolar (1, 3%). Multimedia Appendix 4 provides the characteristics of the included studies, the purposes of the ML techniques used in these studies, and the types of ML models used to diagnose BD in the included studies.

**Table 1:** General characteristics of the included studies (N=33).

Characteristic		Studies, n (%)
**Publication type**		
	Research articles	30 (91)
	Conference proceedings	3 (9)
**Publication status**		
	Published	33 (100)
**Country of publication**		
	China	8 (24)
	United States	7 (21)
	United Kingdom	3 (9)
	Canada	2 (6)
	Germany	2 (6)
	Brazil	1 (3)
	Japan	1 (3)
	Australia	1 (3)
	Italy	1 (3)
	Turkey	1 (3)
	Korea	2 (6)
	Norway	1 (3)
	Netherlands	1 (3)
	India	1 (3)
	Egypt	1 (3)
**Year of publication**		
	2021	6 (18)
	2020	5 (15)
	2019	7 (21)
	2018	7 (21)
	2017	3 (9)
	2016	5 (15)
**Basic purpose**		
	Model development	24 (73)
	Evaluation	5 (15)
	Data analysis	3 (9)
	Model adaptation	2 (6)
**Disorder type**		
	Bipolar disorder type 1	27 (82)
	Bipolar disorder type 2	27 (82)
	Psychotic bipolar	3 (9)
	Chronic bipolar	2 (6)
	First episode bipolar	1 (3)
**Area**		
	Machine learning	33 (100)
	Deep learning	3 (9)
**Purpose of machine learning algorithms**		
	Diagnosis and detection	33 (100)

### Types of ML Models Used to Diagnose Bipolar Disorder in the Included Studies

As shown in Multimedia Appendix 4, the included studies demonstrate 8 types of ML models that have been used to diagnose BD. The most common ML model used belonged to the classification model category, comprising 56% (18/33) of the studies [14,18-26,29-32,36-38,42]. The least commonly used models were natural language processing models [48], clustering algorithms [27], and deep learning–based models [29,30,32]; the various types of models and methods used in the included studies for diagnosing BD are presented in Table 2 and Multimedia Appendix 4.

**Table 2:** Machine learning models and algorithms, methods, and tools used in the included studies (N=33).^a,b^

Model categories		Number of studies, n (%)	Study ID
**Classification models**			
	Support vector machine	9 (28)	[18-26]
	Artificial neural network	4 (12.12)	[29-32]
	Artificial neural network-particle swarm optimization	1 (3.03)	[31]
	Random forest	4 (12.12)	[14,23,29,36]
	Prediction rule ensembles	1 (3.03)	[35]
	Gaussian process model	2 (6.06)	[37,38]
	Nearest neighbor classification algorithm	1 (3.03)	[42]
	Naive Bayes algorithm	1 (3.03)	[42]
	Decision tree algorithm	1 (3.03)	[42]
**Model-based clustering**			
	Growth mixture modeling	1 (3.03)	[41]
	Linear discriminant analysis	1 (3.03)	[19]
**Regression models**			
	Baseline logistic regression	1 (3.03)	[14]
	Linear regression	3 (9.09)	[33,34,47]
	Elastic net method	2 (6.06)	[33,47]
	Least absolute shrinkage and selection operator	2 (6.06)	[19,34]
Fuzzy TOPSIS method		1 (3.03)	[39]
**Clustering algorithms**			
	K-means clustering	1 (3.03)	[27]
**Deep learning–based models**			
	Deep neural network	2 (6.06)	[29,30]
	Convolutional neural network	1 (3.03)	[29]
	DeepBipolar	1 (3.03)	[32]
**Natural language–based model**			
	Natural language processing	1 (3.03)	[48]
**Bipolar disorder assessment tools^c^**			
	Structured clinical interview for DSM-IV^d^	1 (3.03)	[33]

^a^Machine learning models/algorithms were not reported in 2 studies, of which 1 study used a novel machine learning approach to diagnose bipolar disorder type I. The name of the model is not mentioned.

^b^Machine learning methods were only reported in 8 studies.

^c^This is an interview-based assessment tool for diagnosis.

^d^DSM-IV: *Diagnostic and Statistical Manual of Mental Disorders*, Fourth Edition.

#### Classification Models

The includes studies employed 9 different types of classification models. In 9 (28%) of the 33 studies, SVM-based models were used to diagnose BD (specific types are not mentioned) [18-26]. In 1 study [18], this model was used to diagnose chronic BD and first-episode BD, whereas in 3 studies [19,21,26], SVM was used to diagnose type 1 and type 2 BD. However, SVM [24] was also used to diagnose unspecified types of BD. There are 4 studies (12%) that used artificial neural networks (ANNs) [29-32] for diagnosis purposes. Specifically, random forests were used in 4 studies (12%) [14,23,29,36] for diagnosing type 1 and type 2 BD, whereas in 2 studies (6%), Gaussian process models were used to diagnose BD type 1 [37,38]. ANN-particle swarm optimization (ANN-PSO) (3.03%) [31] was only used in 1 study to diagnose BD (types are not mentioned), whereas prediction rule ensembles (3.03%) [35], the decision tree algorithm (3.03%) [42], the nearest neighbor classification algorithm (K-NN) (3.03%) [42], and the naive Bayes algorithm (3.03%) [42] were employed to diagnose type 1 and type 2 BD.

#### Regression Models

The 33 included studies used 4 different types of regression models. Baseline logistic regression used in only 1 (3.03%) study for diagnosing BD and other psychiatric disorders [14]. Linear regression models were used in 3 (9.09%) studies [33,34,47] to diagnose type 1, type 2, and unspecified BD. In 2 (6.06%) studies [33,47], the elastic net method and least absolute shrinkage and selection operator (LASSO) [19,34] were used for diagnosing of type I, type II, and other unspecified BD types.

#### Model-Based Clustering

Linear discriminant analysis (LDA) and growth mixture modeling (GMM) were employed in 2 (6.06%) studies [19,41] for diagnosing type 1 and type 2 BD.

#### Deep Learning–Based Models

Among the 33 studies, 1 (3.03%) used deep neural networks and convolutional Neural Network algorithms [29], and 1 (3.03%) study employed DeepBipolar [32] to diagnose BD (types are not mentioned).

#### Natural Language–Based Model

A natural language processing model was employed by 1 (3.03%) study [48] to diagnose type 1 and type 2 BD.

#### BD Assessment Tools

Only 1 (3.03%) study [33] used SCID (Structured Clinical Interview for DSM-IV), a BD assessment tool, for diagnosing type 1 and type 2 BD.

#### Fuzzy TOPSIS Method

The Fuzzy TOPSIS method was employed in 1 (3.03%) study [39] for diagnosing type 1 and type 2 BD (3.03%).

#### Clustering Algorithms

In 1 study (3.03%) [27], K-means clustering was used for detecting psychotic BD.

### Features of the Data Used in the Included Studies

The sample sizes were not consistent, and different sample sizes were used in the included articles ranging from 15 to 25,000. In 18 (56%) of the 33 studies, the sample size was less than 300 (56%), whereas in 12 (36.4%) studies, the sample size was above 300, as indicated in Table 3 and Multimedia Appendix 4. The most important feature of the included study was the data type. Multidimensional data were used in the selected articles, out of which data in 61.13% (19) of the studies belong to the clinical category, whereas 38.7 % (12) of the studies involved nonclinical data such as that in genomic and genome-wide association studies (GWAS). Private data sources (nongovernment sources or any other clinical data that are not publicly available) were the most commonly used in the included studies, whereas the least commonly used data sources were public (government sources, public databases, online websites, and freely available databases). Most of the included studies used already existing ML models for data evaluation (10, 30.3%), whereas the second common purpose was model adaptation (6, 18.2%). Only few studies developed novel ML models (2, 0.6%), as shown in Multimedia Appendix 4. The most common BD types mentioned in the selected studies were type 1 and type 2, whereas the least common types were chronic bipolar, first episode bipolar, and psychotic bipolar disorders, as observed in Table 1 and Multimedia Appendix 4.

**Table 3:** Features of data used in the included studies (N=33).

Feature		Value
**Data set size (sample size),^a^ n (%)**		
	<100	9 (28)
	100-200	9 (28)
	200-600	7 (21)
	700-1000	3 (9)
	>2000	2 (6)
**Data type,^b^ n (%)**		
	Clinical data	19 (58)
	Nonclinical data	12 (36)
**Data sources,^c^ n (%)**		
	Private	21 (64)
	Public	9 (28)
**Sample type^d^ (%)**		
	Disorder samples	>90
	Healthy control	10

^a^Data set size was only reported in 30 studies.

^b^Data types were only mentioned in 31 studies. Clinical data include blood samples, electronic medical records, neurological data, magnetic resonance imaging data, electroencephalography and microarray expression data, whereas nonclinical data include phenotype data, genotype data, genomic data, and genome wide association studies.

^c^Public data include government sources, public databases, websites, and freely available databases, whereas private data include nongovernment sources, personal information, or data of specific hospitals or research organizations. Private data include databases that are not available in the public domain.

^d^More than 90% of the samples used in the included studies were bipolar disorder samples (regardless of type), whereas 10% of the samples were healthy control samples.

### Types of Data Sets Used in the Included Studies

Data types were only mentioned in the 31 of the 33 studies. As shown in Table 4 and Multimedia Appendix 4, clinical and nonclinical data are reported in the included articles. The following data sets were collected from various public and private sources: clinical data (immune-inflammatory signature, blood sample, neuropsychological, neurocognitive, electroencephalography and PGBI-10M manic symptom data) [18-28,30,31,33,38,41-43,46,49] and nonclinical data (Cambridge Neuropsychological Test Automated Battery cognitive scores, microarray expression data sets, large-scale GWAS, fractional anisotropy, axial diffusivity, radial diffusivity, electronic medical records, bipolarity indices, affective disorder evaluation scale, daily mood ratings survey and phenotypic data sets) [14,19,21,24,29,32,34,36,37,45,47,49,50].

**Table 4:** Data set types used in the included studies (N=33).

Data type^a^			Study reference
**Clinical data (n=19)**			
	Immune-inflammatory signature	[33]	
	Blood samples (serum)	[46]	
	Neuropsychological data	[18]	
	Neurocognitive data	[43]	
	Affective Disorder Evaluation scale	[19]	
	Magnetic resonance imaging ( structural and functional)	[20-23,26-28,30,38,40]	
	Electroencephalography	[24,31]	
	PGBI-10M^b^ manic symptom data	[41]	
	Microarray expression data set	[42]	
**Nonclinical data (n=12)**			
	CANTAB^c^ cognitive scores	[34]	
	Large-scale genome-wide association	[14,45]	
	Phenotypic data set	[36,45,50]	
	Fractional anisotropy	[37]	
	Radial diffusivity	[37]	
	Axial diffusivity	[37]	
	Electronic medical record	[47]	
	Passive digital phenotypes	[36]	
	Bipolarity index	[19]	
	Daily mood ratings survey	[49]	
	Diffusion tensor images	[21,25]	
	Affective Disorder Evaluation scale	[19]	
	Activity monitoring	[29]	
	Genomic data	[31]	

^a^In several studies, more than one data type was used.

^b^PGBI-10M: Parent General Behavior Inventory-10-Item Mania Scale.

^c^CANTAB: Cambridge Neuropsychological Test Automated Battery.

### Statistical Validations of ML Models and Algorithms, Methods, and Tools Used in the Included Studies

The accuracies of the ML models and algorithms were reported in 24 studies, as shown in Table 5 and Multimedia Appendix 4. The accuracy level ranged from ≤70% to >91%. The accuracy level was ≤70% in 3 studies [25,32,36], 71%-78% in 7 studies [18,23,34,37,43,47,49], 83%-90% in 9 studies [14,20,21,26,28,29,31,33,42], and >91% in 5 studies [19,22,24,35,40]. The highest accuracy was 98%, found in only 1 study, whereas the lowest accuracy level was 64%. The mean value of the accuracy in 26 studies was 82.06%, whereas the median value was 84%.

Sensitivity was reported in only 15 studies; it ranged from ≤60% to >90%. Sensitivity was ≤60% in 1 study [46], 65%-67% in 2 studies [37,38], 75%-78% in 3 studies [14,18,47], 80%-88% in 8 studies [20-22,26,29,31,41,42], and above 90% in 1 study [35]. The mean value of sensitivity was 78.26%, whereas the median value was 82%. Moreover, specificity was only mentioned in 13 studies. The value of specificity ranged from ≤70% to 92% in 1 study [46], 74%-77% in 3 studies [18,42,49], 80%-90% in 6 studies [14,20,29,37,41,47], and >90% in 4 studies [21,22,26,35]. The mean specificity value was 85.36% and the median was 85.4%.

The proportion of the area under the curve (AUC) value was only reported in 10 studies, ranging from ≤69% to >97%. In 3 studies, the AUC ratio was ≤70% [23,36,45]; in 2 studies, it was 74%-78% [31,43] and 84%-88% [14,47], and in 3 studies [19,28,33], it was >90%. The maximum AUC ratio was 97%, whereas the minimum AUC value was 65%. The mean AUC value was 80.95% in 10 studies, whereas the median value was 81%.

**Table 5:** Statistical validation.

Statistics		Study reference
**Accuracy, %^a^**		
	≤70	[25,31,36]
	71-78	[18,23,34,37,43,47,49]
	83-90	[14,20,21,26,28,29,31,33,42]
	>91	[19,22,24,35,40]
**Sensitivity, %^b^**		
	≤60	[46]
	65-67	[37,38]
	75-78	[14,18,47]
	80-88	[20-22,26,29,31,41,43]
	>90	[35]
**Specificity, %^c^**		
	≤70	[46]
	74-77	[18,38,43]
	81-89	[14,20,29,37,41,47]
	>92	[21,22,26,35]
**AUC, %^d^**		
	≤70	[23,36,45]
	74-78	[32,43]
	84- 88	[14,47]
	>91	[19,28,33]

^a^Ratio of accuracy was not reported in 7 studies. In some studies, different values were mentioned, so the overall values do not sum up.

^b^Sensitivity was not mentioned in 18 studies.

^c^Specificity was not mentioned in 20 studies.

^d^AUC: area under the curve. It is basically used for statistical validation of any data. AUC values were not reported in 23 studies.

## Discussion

### Principal Findings

Previous studies stressed the importance of ML classifiers to aid in diagnosing BD accurately, as it is frequently misdiagnosed. Approximately 60% of BD cases are misdiagnosed as major depressive disorders, and a proper diagnosis may take up to 10 years [46]. AI and ML exhibit considerable potential in clinical decision support and analysis with the help of big data, especially in mental health [7].

In this review, we explored the uses of ML techniques in diagnosing BD. From the 573 studies retrieved, 33 studies were included in this review. To explore the use of ML in diagnosing BD, the information was classified into 3 main categories as follows:

### Machine Learning Models Used for Diagnosing BD

This review identified ML models, methods, and tools used for diagnosing BD, some of which did not use ML methods as the primary tool for diagnosis but used them as a supportive tool.

SVMs were the most commonly used ML models in diagnosing BD in 9 (27%) of the 33 studies, followed by ANNs (5, 15%), followed ensemble models (3, 9%), linear regression (3, 9%), and the Gaussian process model (2, 6%). Further, natural language processing, linear discriminant analysis, and logistic regression were used once in each study (3, 9%). Additionally, 7 studies applied other ML models that were emerging models or used a program to perform the diagnoses. However, only 1 study used a BD assessment tool, SCID, for the diagnosis of BD and an ML model as a supportive tool. Further, 1 study did not specify which ML model was employed. Hence, the use of ML models to diagnose BD is influenced by the diagnosis of BD, which is why studies have been exploring different ML models to better diagnose such mental disorders.

### Data Sets Used in the Included Studies

The included studies used 2 types of data in diagnosing BD (clinical and nonclinical data). Clinical data were the most widely used, in 19 (53%) of the 33 studies. Among these 19 studies, 10 used magnetic resonance imaging (MRI) to classify bipolar patients compared to other groups. Other less commonly used data are mentioned in Table 4.

Nonclinical data were used in 12 studies (36%); some examples of nonclinical data used are large-scale GWAS (2, 6%), phenotypic data sets (2, 6%), diffusion tensor images (DTIs) (2, 6%) and other less commonly used data (Table 4). It is not surprising that nonclinical data are less commonly used because they mainly depend on surveys and tests related to mental disorders, which may lead to some biased results.

### Validation of ML Models

The retrieved studies used 4 main validation measures to validate the ML models; these measures are accuracy, sensitivity, specificity, and AUC.

The accuracy of the ML models and algorithms was reported in 24 studies. The accuracy ranged from ≤70% to >91%. The highest accuracy achieved was 98% in only 1 study, whereas the lowest accuracy was 64%. Most studies achieved an accuracy of 83%-90% (9, 37.5%). The mean value of the accuracy was 82.06%. Moreover, sensitivity was only reported in 15 studies; it ranged from ≤60 to >90%. The mean value of sensitivity was 78.26%, whereas most studies (8, 53.3%) achieved sensitivity values between 80% and 88%. Furthermore, specificity was only mentioned in 13 studies. The value of specificity ranged from ≤70 to 92%. The mean value of Specificity was 85.4%, and most studies (6, 46.15%) achieved specificity values of 80%-90%. Finally, the AUC value was only reported in 10 studies, ranging from ≤69% to >97%. The maximum AUC value was 97%, whereas the minimum value was 65%. The mean AUC value was 81%. An important factor is that we were unable to compare the ML models and better categorize them owing to the variety of validation methods used in the reviewed studies. However, accuracy tended to be the most used measure in validating the ability of ML models to diagnose BD.

### Comparison With Prior Work

Diego et al [10] conducted a systematic review that explored the applications of ML in diagnosing BD. The authors included articles from PubMed, Embase, and Web of Science published in any language up to 2017. They extracted 757 articles and included 51 studies in their review. They focused on categorizing the studies based on the data used to diagnose, treat, and prevent BD. Our focus was providing insight on the ML techniques used to diagnose various types of BD, including bipolar 1, bipolar 2, chronic bipolar, and episode bipolar. However, the articles lack information on the type of BD used to train and test the ML models (20 out of 33 studies did not specify the BD type). Thus, the data were categorized based on the ML model used to classify bipolar patients. Furthermore, we highlighted the advantages of the different data types used for different ML models. MRI data that were specifically used for SVMs and Gaussian process models showed good accuracy. However, EEG data used for SVMs showed higher accuracy than MRI data (98%), whereas DTI data showed lower accuracy than MRI and EEG data in case of SVMs (68.3%). Hence, we can infer that the predictive power and accuracy of ML models depend on the type of input data, as summarized in Table 6.

**Table 6:** Model performance metrics.

Data type	Study ID	Proposed model	Sensitivity, %	Specificity, %	Accuracy, %	AUC^a^
GWAS^b^	[14]	Random forest	77.7	85.4	85.2	NR^c^
Neuropsychological data	[18]	SVM^d^	76	77	77.0	NR
ADE^e^ and BPx^f^	[19]	SVM	NR	NR	96.0	92.1
MRI^g^	[20]	SVM	85	85	85	NR
MRI	[21]	SVM	82.3	92.7	87.6	NR
MRI	[22]	SVM	87.5	97.1	92.4	NR
MRI	[23]	SVM	NR	NR	76.0	74
MRI	[26]	SVM	84.6	92.3	83.5	NR
MRI	[38]	Gaussian process model	66.4	74.2	70.3	NR
EEG^h^	[24]	SVM	NR	NR	98.0	NR
	[31]	ANN^i^	83.87	NR	89.89	NR
DTI^j^	[25]	SVM	NR	NR	68.3	NR
Activity monitoring	[29]	RF,^k^ CNN,^l^ and ANN	82	84	84	NR
Genomic data	[31]	ANN-PSO^m^	83.87	NR	89.89	NR
Immune-inflammatory signature	[33]	Linear regression and elastic net methods	NR	NR	86	97
EMR^n^	[47]	Linear regression and elastic net methods	75	81	78	84
CANTAB^o^ cognitive score	[34]	Linear regression and LASSO^p^	NR	NR	71.0	NR
Phenotypic data set (passive digital phenotype)	[36]	RF	NR	NR	65	67
Fractional anisotropy, radial diffusivity, and axial diffusivity	[37]	Gaussian Process model	66.67	84.21	75.0	NR
PGBI-10M^q^ manic symptom data	[41]	Growth mixture modeling	83	89	NR	NR

^a^AUC: area under the curve.

^b^GWAS: genome-wide association.

^c^NR: not reported in the article.

^d^SVM: support vector machine.

^e^ADE: Affective Disorder Evaluation.

^f^BPx: bipolarity index.

^g^MRI: magnetic resonance imaging.

^h^EEG: electroencephalography.

^i^ANN: artificial neural network.

^j^DTI: diffusion tensor images.

^k^RF: random forest.

^l^CNN: convolutional neural network.

^m^ANN-PSO: ANN-particle swarm optimization.

^n^EMR: electronic medical record.

^o^CANTAB: Cambridge Neuropsychological Test Automated Battery.

^p^LASSO: least absolute shrinkage and selection operator.

^q^PGBI-10M: Parent General Behavior Inventory-10-Item Mania Scale.

### Future Research and Practical Implications

This review categorized the most common ML models and data used in diagnosing BD. Based on our findings, ML models can diagnose BD using clinical and nonclinical data. Future research should explore the studies involving patients in clinical and nonclinical settings to better evaluate the accuracy of the ML models.

Moreover, future studies should explore the influence of external factors like social media and the influence of the society on mental disorders to evaluate the influence of these factors on the patients and their effects on the performance of the ML models.

Furthermore, ML models should be compared with other traditional techniques for diagnosing BD like the Affective Disorder Evaluation (ADE) scale and Structured Clinical Interview for DSM-IV.

Only 2 studies reviewed used data sets with sizes above 2000, which is not surprising considering that most studies had data size as a limitation. In future studies, the ML models should be trained and validated on a larger data set and have a larger healthy control sample, as it was less than 10% in the reviewed studies.

As AI use in the health sector is growing rapidly, physicians should pay careful attention to some major issues that stand in the way of dealing with sensitive data such as medical information because of data ownership and security issues.

BD symptoms overlap with other mood disorders, specifically MDD, and this leads to the misdiagnosis of BD [20]. Future research should explore the main indicator that shows the patient is diagnosed with BD; for example, studies showed that patients diagnosed with BD have abnormal gray matter density in the MRI images of the brain. Another major indicator is regional homogeneity (ReHo), which indicates the activity of the brain while at rest [20,23]. Although some studies explored the use of some ML techniques that use binary classification methods such as SVMs and logistic regression, it is still not clear how ML techniques can distinguish BD, healthy people, and other mood disorders without the need for 2 groups (binary classification).

In addition, clinicians and researchers should explore the use of ML technology in clinical settings and address the clinical implications and outcomes of ML in diagnosing BD. Future investigations should focus on understanding of people’s physiological and psychological behavior regarding the use of these technologies and the level of acceptance shown by physicians and patients. Finally, clinicians should explore the effectiveness of diagnosing models in clinical settings and develop predictive models that can predict mental disorders like BD.

### Strengths and Limitations

#### Strengths

The present review was conducted to address the lack of scoping reviews that gather and categorize ML models used in diagnosing BD. The importance of this review stems from the fact that the traditional ways of diagnosing BD may lead to late diagnosis (an average of 10 years delay until formal diagnosis). This review explored studies that examined the ability of ML models to diagnose BD using a variety of data.

The most recent reviews focused on the implications of ML in patients with BD focused either on a specific ML model (neural networks) [51] or on the application of ML using MRI data [52]. This review explored the application of ML models in diagnosing BD without any limitations in terms of the technique or the type of data used, which gives a deeper insight into the technologies used in this field.

The studies considered in this review were the latest one to reduce bias in terms of date selection. We also conducted a backward referencing check by which we found 2 studies. Finally, the study selection included 3 reviewers working independently and any disagreements in the process were discussed and a decision was made upon consensus; this ensured reduced selection bias.

#### Limitations

This review included only 3 databases (PubMed, Google Scholar, and ScienceDirect), and other databases were not included, such as Embase, IEEE, Scopus, and the ACM Digital Library. This may have led to the absence of some studies that might be relevant to our review; for example, we did not include XGBoosting or LGBM, which are the most common ensemble models used for diagnosis purposes. Some of these databases were not included because of inaccessibility and time constraints. Moreover, we only considered articles published in the last 5 years (2016-2021). We missed categorizing supervised and unsupervised ML models, such as logistic regression, which is a supervised learning method.

We retrieved studies published in English only, which potentially led to the absence of other relevant studies published in other languages, especially French. Our study included data belonging to the United States, United Kingdom, China, Germany, Japan, Turkey, Korea, Italy, India, Canada, Norway, Egypt, Australia, Brazil, and the Netherlands. We missed including data from other populations. This made our results less comprehensive.

Furthermore, this review focused mainly on ML models diagnosing BD, regardless of what the patients were compared to in the training and testing sets (other psychiatric diagnoses) and regardless of the demographics of the patients. This may lead to biased decisions compared to other psychiatric diagnoses without having a healthy control sample. Moreover, our search queries lacked terms related to specific ML algorithms or models. Hence, we did not retrieve articles that used these terms in the title or abstract instead of ML. This again reduced the diversity of our scoping review.

### Conclusions

This scoping review grouped recent studies based on the ML model used to diagnose patients with BD regardless of their demographics or their assessments compared to patients with other psychiatric diagnoses. We have also provided information about the data used and summarized the data that were most commonly used in diagnosing BD. The goal of this review was to provide insights into how these technologies can help in faster and better diagnosis of BD and to promote their use in making clinical decisions in the health industry.

## Appendix

Multimedia Appendix 1 List of queries used in various databases.

Multimedia Appendix 2 Description of data extraction fields.

Multimedia Appendix 3 Characteristics of the included studies and purposes of machine learning techniques used in the studies.

Multimedia Appendix 4 Summary of all the data extracted from the included studies.

Multimedia Appendix 5 Fractions of articles by publication type.

Multimedia Appendix 6 Fractions of numbers of articles published by year.

## Abbreviations

AD: axial diffusivity
ADE: Affective Disorder Evaluation
AI: artificial intelligence
ANN-PSO: Artificial neural network-particle swarm optimization
BD: bipolar disorder
CANTAB: Cambridge Neurophysiological Test Automated Battery
DTI: diffusion tensor images
EEG: electroencephalography
EHR: electronic health record
FA: fractional anisotropy
fMRI: functional magnetic resonance imaging
GPC: Gaussian process classifier
GWA: genome-wide association data
LR: logistic regression
ML: machine learning
MRI: magnetic resonance imaging
NLP: natural language processing
OCD: obsessive compulsive disorder
PRISMA-ScR: Preferred Reporting Items for Systematic Reviews and Meta-Analyses Extension for Scoping Reviews
RD: radial diffusivity
RF: random forest
rs-fMRI: resting state functional magnetic resonance imaging
SVM: support vector machine
YBOCS: yellow-brown obsessive-compulsive disorder

## References

[1] Passos IC, Ballester Pedro L, Barros Rodrigo C, Librenza-Garcia Diego, Mwangi Benson, Birmaher Boris, Brietzke Elisa, Hajek Tomas, Lopez Jaramillo Carlos, Mansur Rodrigo B, Alda Martin, Haarman Bartholomeus C M, Isometsa Erkki, Lam Raymond W, McIntyre Roger S, Minuzzi Luciano, Kessing Lars V, Yatham Lakshmi N, Duffy Anne, Kapczinski Flavio (2019). Machine learning and big data analytics in bipolar disorder: a position paper from the International Society for Bipolar Disorders Big Data Task Force. Bipolar Disord.

[2] Marrie R, Walld R, Bolton J, Sareen J, Walker J, Patten S, Singer A, Lix L, Hitchon C, El-Gabalawy R, Katz A, Fisk John D, Bernstein Charles N, CIHR Team in Defining the Burden and Managing the Effects of Psychiatric Comorbidity in Chronic Immunoinflammatory Disease (2017). Increased incidence of psychiatric disorders in immune-mediated inflammatory disease. J Psychosom Res.

[3] Dome P, Rihmer Z, Gonda X (2019). Suicide risk in bipolar disorder: a brief review. Medicina (Kaunas).

[4] Navarro-Mateu F, Alonso J, Lim CCW, Saha S, Aguilar-Gaxiola S, Al-Hamzawi A, Andrade LH, Bromet EJ, Bruffaerts R, Chatterji S, Degenhardt L, de Girolamo G, de Jonge P, Fayyad J, Florescu S, Gureje O, Haro JM, Hu C, Karam EG, Kovess-Masfety V, Lee S, Medina-Mora ME, Ojagbemi A, Pennell B, Piazza M, Posada-Villa J, Scott KM, Stagnaro JC, Xavier M, Kendler KS, Kessler RC, McGrath JJ, WHO World Mental Health Survey Collaborators (2017). The association between psychotic experiences and disability: results from the WHO World Mental Health Surveys. Acta Psychiatr Scand.

[5] Yatham LN, Kennedy SH, Parikh SV, Schaffer A, Bond DJ, Frey BN, Sharma V, Goldstein BI, Rej S, Beaulieu S, Alda M, MacQueen G, Milev RV, Ravindran A, O'Donovan C, McIntosh D, Lam RW, Vazquez G, Kapczinski F, McIntyre RS, Kozicky J, Kanba S, Lafer B, Suppes T, Calabrese JR, Vieta E, Malhi G, Post RM, Berk M (2018). Canadian Network for Mood and Anxiety Treatments (CANMAT) and International Society for Bipolar Disorders (ISBD) 2018 guidelines for the management of patients with bipolar disorder. Bipolar Disord.

[6] Staudt Hansen P, Frahm Laursen M, Grøntved Simon, Puggard Vogt Straszek S, Licht RW, Nielsen RE (2019). Increasing mortality gap for patients diagnosed with bipolar disorder-A nationwide study with 20 years of follow-up. Bipolar Disord.

[7] Malhi G, Bell E (2020). Prepubertal bipolar disorder: a diagnostic quandary?. Int J Bipolar Disord.

[8] Lin E, Lin C, Lane H (2020). Precision psychiatry applications with pharmacogenomics: artificial intelligence and machine learning approaches. Int J Mol Sci.

[9] Fernandes BS, Karmakar C, Tamouza R, Tran T, Yearwood J, Hamdani N, Laouamri H, Richard J, Yolken R, Berk M, Venkatesh S, Leboyer M (2020). Precision psychiatry with immunological and cognitive biomarkers: a multi-domain prediction for the diagnosis of bipolar disorder or schizophrenia using machine learning. Transl Psychiatry.

[10] Librenza-Garcia D, Kotzian BJ, Yang J, Mwangi B, Cao B, Pereira Lima LN, Bermudez MB, Boeira MV, Kapczinski F, Passos IC (2017). The impact of machine learning techniques in the study of bipolar disorder: a systematic review. Neurosci Biobehav Rev.

[11] Kessing LV, Andersen PK, Vinberg M (2018). Risk of recurrence after a single manic or mixed episode - a systematic review and meta-analysis. Bipolar Disord.

[12] Huang Y, Wang Y, Wang H, Liu Z, Yu X, Yan J, Yu Y, Kou C, Xu X, Lu J, Wang Z, He S, Xu Y, He Y, Li T, Guo W, Tian H, Xu G, Xu X, Ma Y, Wang L, Wang L, Yan Y, Wang B, Xiao S, Zhou L, Li L, Tan L, Zhang T, Ma C, Li Q, Ding H, Geng H, Jia F, Shi J, Wang S, Zhang N, Du X, Du X, Wu Y (2019). Prevalence of mental disorders in China: a cross-sectional epidemiological study. Lancet Psychiatry.

[13] Jie N, Zhu M, Ma X, Osuch E, Wammes M, Theberge J, Li H, Zhang Y, Jiang T, Sui J, Calhoun V (2015). Discriminating bipolar disorder from major depression based on SVM-FoBa: efficient feature selection with multimodal brain imaging data. IEEE Trans Auton Mental Dev.

[14] Chuang L, Kuo P (2017). Building a genetic risk model for bipolar disorder from genome-wide association data with random forest algorithm. Sci Rep.

[15] Munn Z, Peters M, Stern C, Tufanaru C, McArthur A, Aromataris E (2018). Systematic review or scoping review? Guidance for authors when choosing between a systematic or scoping review approach. BMC Med Res Methodol.

[16] Tricco A, Lillie E, Zarin W, O'Brien K, Colquhoun H, Levac D, Moher D, Peters M, Horsley T, Weeks L, Hempel S (2018). PRISMA extension for scoping reviews (PRISMA-ScR): checklist and explanation. Ann Intern Med.

[17] (2021). Rayyan Systems.

[18] Sawalha J, Cao L, Chen J, Selvitella A, Liu Y, Yang C, Li X, Zhang X, Sun J, Zhang Y, Zhao L, Cui Liqian, Zhang Yizhi, Sui Jie, Greiner Russell, Li Xin-Min, Greenshaw Andrew, Li Tao, Cao Bo (2021). Individualized identification of first-episode bipolar disorder using machine learning and cognitive tests. J Affect Disord.

[19] Ma Y, Ji J, Huang Y, Gao H, Li Z, Dong W, Zhou S, Zhu Y, Dang W, Zhou T, Yu H, Yu B, Long Y, Liu L, Sachs G, Yu X (2019). Implementing machine learning in bipolar diagnosis in China. Transl Psychiatry.

[20] Li H, Cui L, Cao L, Zhang Y, Liu Y, Deng W, Zhou W (2020). Identification of bipolar disorder using a combination of multimodality magnetic resonance imaging and machine learning techniques. BMC Psychiatry.

[21] Achalia R, Sinha A, Jacob A, Achalia G, Kaginalkar V, Venkatasubramanian G, Rao NP (2020). A proof of concept machine learning analysis using multimodal neuroimaging and neurocognitive measures as predictive biomarker in bipolar disorder. Asian J Psychiatr.

[22] Osuch E, Gao S, Wammes M, Théberge J, Willimason P, Neufeld RJ, Du Y, Sui J, Calhoun V (2018). Complexity in mood disorder diagnosis: fMRI connectivity networks predicted medication-class of response in complex patients. Acta Psychiatr Scand.

[23] Schwarz E, Doan N, Pergola G, Westlye L, Kaufmann T, Wolfers T, Brecheisen R, Quarto T, Ing A, Di Carlo Pasquale, Gurholt T, Harms Robbert L, Noirhomme Quentin, Moberget Torgeir, Agartz Ingrid, Andreassen Ole A, Bellani Marcella, Bertolino Alessandro, Blasi Giuseppe, Brambilla Paolo, Buitelaar Jan K, Cervenka Simon, Flyckt Lena, Frangou Sophia, Franke Barbara, Hall Jeremy, Heslenfeld Dirk J, Kirsch Peter, McIntosh Andrew M, Nöthen Markus M, Papassotiropoulos Andreas, de Quervain Dominique J-F, Rietschel Marcella, Schumann Gunter, Tost Heike, Witt Stephanie H, Zink Mathias, Meyer-Lindenberg Andreas, IMAGEMEND Consortium‚ Karolinska Schizophrenia Project (KaSP) Consortium (2019). Reproducible grey matter patterns index a multivariate, global alteration of brain structure in schizophrenia and bipolar disorder. Transl Psychiatry.

[24] El Gohary MI, Al Zohairy TA, Eissa AM, El Deghaidy S, Hussein HM (2016). An intelligent system for diagnosis of schizophrenia and bipolar diseases using support vector machine with different kernels. Int J Appl Eng Sci.

[25] Deng F, Wang Y, Huang H, Niu M, Zhong S, Zhao L, Qi Z, Wu X, Sun Y, Niu C, He Y, Huang L, Huang R (2018). Abnormal segments of right uncinate fasciculus and left anterior thalamic radiation in major and bipolar depression. Prog Neuropsychopharmacol Biol Psychiatry.

[26] Frangou S, Dima D, Jogia J (2017). Towards person-centered neuroimaging markers for resilience and vulnerability in bipolar disorder. Neuroimage.

[27] Mothi SS, Sudarshan M, Tandon N, Tamminga C, Pearlson G, Sweeney J, Clementz B, Keshavan MS (2019). Machine learning improved classification of psychoses using clinical and biological stratification: update from the bipolar-schizophrenia network for intermediate phenotypes (B-SNIP). Schizophr Res.

[28] Liu Y, Mellem MS, Gonzalez H, Kollada M, Mahableshwarkar AR, Madrid A, Martin WJ, Ahammad P Highly predictive transdiagnostic features shared across schizophrenia, bipolar disorder, and adhd identified using a machine learning based approach. bioRxiv.

[29] Jakobsen P, Garcia-Ceja E, Riegler M, Stabell L, Nordgreen T, Torresen J, Fasmer O, Oedegaard K (2020). Applying machine learning in motor activity time series of depressed bipolar and unipolar patients compared to healthy controls. PLoS One.

[30] Gong J, Chen G, Jia Y, Zhong S, Zhao L, Luo X, Qiu S, Lai S, Qi Z, Huang L, Wang Y (2019). Disrupted functional connectivity within the default mode network and salience network in unmedicated bipolar II disorder. Prog Neuropsychopharmacol Biol Psychiatry.

[31] Erguzel T, Sayar G, Tarhan N (2015). Artificial intelligence approach to classify unipolar and bipolar depressive disorders. Neural Comput Appl.

[32] Sundaram Laksshman, Bhat RR, Viswanath V, Li X (2017). DeepBipolar: Identifying genomic mutations for bipolar disorder via deep learning. Hum Mutat.

[33] Poletti S, Vai B, Mazza MG, Zanardi R, Lorenzi C, Calesella F, Cazzetta S, Branchi I, Colombo C, Furlan R, Benedetti F (2021). A peripheral inflammatory signature discriminates bipolar from unipolar depression: a machine learning approach. Prog Neuropsychopharmacol Biol Psychiatry.

[34] Wu M, Passos IC, Bauer IE, Lavagnino L, Cao B, Zunta-Soares GB, Kapczinski F, Mwangi B, Soares JC (2016). Individualized identification of euthymic bipolar disorder using the Cambridge Neuropsychological Test Automated Battery (CANTAB) and machine learning. J Affect Disord.

[35] Parker G, Spoelma MJ, Tavella G, Alda M, Hajek T, Dunner DL, O'Donovan C, Rybakowski JK, Goldberg JF, Bayes A, Sharma V, Boyce P, Manicavasagar V (2021). Differentiating mania/hypomania from happiness using a machine learning analytic approach. J Affect Disord.

[36] Cho C, Lee T, Kim M, In HP, Kim L, Lee H (2019). Mood prediction of patients with mood disorders by machine learning using passive digital phenotypes based on the circadian rhythm: prospective observational cohort study. J Med Internet Res.

[37] Linke JO, Adleman NE, Sarlls J, Ross A, Perlstein S, Frank HR, Towbin KE, Pine DS, Leibenluft E, Brotman MA (2020). White matter microstructure in pediatric bipolar disorder and disruptive mood dysregulation disorder. J Am Acad Child Adolesc Psychiatry.

[38] Mwangi B, Wu M, Cao B, Passos IC, Lavagnino L, Keser Z, Zunta-Soares GB, Hasan KM, Kapczinski F, Soares JC (2016). Individualized prediction and clinical staging of bipolar disorders using neuroanatomical biomarkers. Biol Psychiatry Cogn Neurosci Neuroimaging.

[39] Han Y, Lu Z, Du Z, Luo Q, Chen S (2018). A YinYang bipolar fuzzy cognitive TOPSIS method to bipolar disorder diagnosis. Comput Methods Programs Biomed.

[40] Palaniyappan L, Deshpande G, Lanka P, Rangaprakash D, Iwabuchi S, Francis S, Liddle PF (2019). Effective connectivity within a triple network brain system discriminates schizophrenia spectrum disorders from psychotic bipolar disorder at the single-subject level. Schizophr Res.

[41] Jo B, Findling RL, Hastie TJ, Youngstrom EA, Wang C, Arnold LE, Fristad MA, Frazier TW, Birmaher B, Gill MK, Horwitz SM (2018). Construction of longitudinal prediction targets using semisupervised learning. Stat Methods Med Res.

[42] Saylan C, Yilancioglu K (2016). Classification of schizophrenia and bipolar disorder by using machine learning algorithms. J Neurobehav Sci.

[43] Sonkurt HO, Altınöz Ali Ercan, Çimen Emre, Köşger Ferdi, Öztürk Gürkan (2021). The role of cognitive functions in the diagnosis of bipolar disorder: a machine learning model. Int J Med Inform.

[44] Dwyer DB, Falkai P, Koutsouleris N (2018). Machine learning approaches for clinical psychology and psychiatry. Annu Rev Clin Psychol.

[45] Shafquat A, Crystal R, Mezey J (2020). Identifying novel associations in GWAS by hierarchical Bayesian latent variable detection of differentially misclassified phenotypes. BMC Bioinformatics.

[46] Idemoto K, Ishima T, Niitsu T, Hata T, Yoshida S, Hattori K, Horai T, Otsuka I, Yamamori H, Toda S, Kameno Y, Ota Kiyomitsu, Oda Yasunori, Kimura Atsushi, Hashimoto Tasuku, Mori Norio, Kikuchi Mitsuru, Minabe Yoshio, Hashimoto Ryota, Hishimoto Akitoyo, Nakagome Kazuyuki, Iyo Masaomi, Hashimoto Kenji (2021). Platelet-derived growth factor BB: a potential diagnostic blood biomarker for differentiating bipolar disorder from major depressive disorder. J Psychiatr Res.

[47] Suen PJC, Goerigk S, Razza LB, Padberg F, Passos IC, Brunoni AR (2021). Classification of unipolar and bipolar depression using machine learning techniques. Psychiatry Res.

[48] Chandran D, Robbins DA, Chang C, Shetty H, Sanyal J, Downs J, Fok M, Ball M, Jackson R, Stewart R, Cohen H, Vermeulen JM, Schirmbeck F, de Haan L, Hayes R (2019). Use of natural language processing to identify obsessive compulsive symptoms in patients with schizophrenia, schizoaffective disorder or bipolar disorder. Sci Rep.

[49] Perez Arribas I, Goodwin GM, Geddes JR, Lyons T, Saunders KEA (2018). A signature-based machine learning model for distinguishing bipolar disorder and borderline personality disorder. Transl Psychiatry.

[50] Fernandes BS, Karmakar C, Tamouza R, Tran T, Yearwood J, Hamdani N, Laouamri H, Richard J, Yolken R, Berk M, Venkatesh S, Leboyer M (2020). Precision psychiatry with immunological and cognitive biomarkers: a multi-domain prediction for the diagnosis of bipolar disorder or schizophrenia using machine learning. Transl Psychiatry.

[51] Yasin S, Hussain SA, Aslan S, Raza I, Muzammel M, Othmani A (2021). EEG based major depressive disorder and bipolar disorder detection using neural networks: a review. Comput Methods Programs Biomed.

[52] Kim Y, Na K (2018). Application of machine learning classification for structural brain MRI in mood disorders: critical review from a clinical perspective. Prog Neuropsychopharmacol Biol Psychiatry.

