# Supplementary material for: The Role of Machine Learning in Diagnosing Bipolar Disorder: Scoping Review
Source: J Med Internet Res. 2021 Nov 19;23(11):e29749. doi: 10.2196/29749 (PMC8663682; doi:10.2196/29749)
Supplement: Multimedia Appendix 1 [file jmir_v23i11e29749_app1.docx]

**Multimedia Appendix 1**

List of Query used in various databases

| **Database** | **Query** |
| --- | --- |
| **PubMed** | ("Artificial Intelligence*" OR "Deep Learning" OR "Machine Learning" OR "Natural Language Processing" OR neural network* OR "unsupervised learning" OR "supervised learning" "Artificial Intelligence"[MeSH Major Topic] OR "Deep Learning"[MeSH Major Topic] OR "Machine Learning"[MeSH Terms] OR "Supervised Machine Learning"[MeSH Terms] OR "Algorithms"[MeSH Major Topic]) AND ("Bipolar disorder" OR bipolar* OR "Bipolar 1 Disorder" OR "Bipolar 2 Disorder" OR "bipolar mood disorder" OR "bipolar affective disorder" OR "Cyclothymic Disorder" OR Cyclothym* OR "manic depression" OR manic* OR "Bipolar Disorder"[MeSH Terms] OR "Major Affective Disorder 1"[Supplementary Concept] OR "Major Affective Disorder 3"[Supplementary Concept] OR "Cyclothymic Disorder"[MeSH Terms] OR "Mania"[MeSH Terms]) AND (diagnos* OR recog* OR prognosis OR detect* OR screening* OR "Diagnosis"[Mesh] OR "Early Diagnosis"[Mesh] OR "Prognosis"[Majr]) - 121 results. |
| **Google Scholar** | (“Artificial Intelligence*” OR “Deep Learning” OR  “Machine Learning”)  AND (“Bipolar disorder” OR bipolar* OR  “Cyclothymic Disorder” OR Cyclothym*  OR “manic depression” OR manic*) AND  (diagnos* OR recog* OR prognosis OR detect*)  - 14,900 results  (Reduced the number of words as Google scholar takes 30 words only). |
| **ScienceDirect** | ((“Machine Learning” OR “ML” OR “Artificial Intelligence” OR “AI”) AND (“Bipolar Disorder” OR “Bipolar” OR “Cyclothymic Disorder”) 15,567 results) and stop when it is no longer relevant (350 studies). |
