# Supplementary material for: The Role of Machine Learning in Diagnosing Bipolar Disorder: Scoping Review
Source: J Med Internet Res. 2021 Nov 19;23(11):e29749. doi: 10.2196/29749 (PMC8663682; doi:10.2196/29749)
Supplement: Multimedia Appendix 2 [file jmir_v23i11e29749_app2.docx]

**Appendix 2**

**Description of data extraction fields**

| **Data Extraction Field** | **Description** |
| --- | --- |
| **Title** | The title of the article |
| **First Author** | The name of the first author in the article |
| **Year of Publication** | The year of the publication of each article is recorded. |
| **Country** | Country in which article published was noted. |
| **Study level** | Studies were conducted at two level.  1: Population level  2: Individual level |
| **Outcome** | Represent accuracy and statistical validation of the ML models used in the included studies |
| **Data settings** | Represent which type of data utilized by the authors.  Data types was two categories.  1: Clinical  2: Non-clinical |
| **Data Availability** | Represent data are freely available or not.  Two data sources  1: Public-All data used by the author are publicly available  2: Private- Data are not publicly available |
| **Publication Type** | Show whether studies are research articles or Conference Proceedings |
| **Study settings** | ML model used in the study was novel or existing. |
