# Supplementary material for: The Role of Machine Learning in Diagnosing Bipolar Disorder: Scoping Review
Source: J Med Internet Res. 2021 Nov 19;23(11):e29749. doi: 10.2196/29749 (PMC8663682; doi:10.2196/29749)
Supplement: Multimedia Appendix 3 [file jmir_v23i11e29749_app3.docx]

**Appendix 3: Characteristics of the included studies and purpose of ML techniques used in the studies**

| **First author** | **Year** | **Country** | **Purpose of the Machine learning models/algorithms, methods, and tools** | **Publication Type** |
| --- | --- | --- | --- | --- |
| Poletti | 2021 | Italy | Diagnosis of bipolar but types was not specified | Research Article |
| Idemoto | 2021 | Japan | Diagnosis of bipolar but types was not specified | Research Article |
| Suen | 2021 | Brazil & Germany | Diagnosis of BD type I, BD Type II & BD not specified | Conference Proceedings |
| Sawalha | 2021 | China | Diagnose chronic BD and first-episode BD | Research Article |
| Parker | 2021 | Australia | Diagnoses of bipolar I, bipolar II | Research Article |
| Sonkurt | 2021 | Turkey | Bipolar type 1 diagnosis | Research Article |
| Li | 2020 | China | Purpose was to diagnose bipolar, but types was not specified | Research Article |
| Jakobsen | 2020 | Korea | Purpose was to diagnose bipolar, but types was not specified | Research Article |
| Achalia | 2020 | India | BD type I Diagnosis | Research Article |
| Linke | 2020 | Maryland | Diagnoses of Not specified bipolar type | Research Article |
| Shafquat | 2020 | USA | Purpose was to diagnose bipolar, but types was not specified | Research Article |
| Cho | 2019 | Korea | BD type I, BD Type II diagnosis | Research Article |
| Chandran | 2019 | UK | Purpose was to diagnose bipolar, but types was not specified | Research Article |
| Ma | 2019 | China | Purpose was to diagnose bipolar, but types was not specified | Research Article |
| Gong | 2019 | China | BD type II diagnosis | Research Article |
| Schwarz | 2019 | Germany | Purpose was to diagnose bipolar, but types was not specified | Research Article |
| Palaniyappan | 2019 | Canada | Psychotic bipolar disorder diagnosis | Research Article |
| Mothi | 2019 | USA | Purpose was to diagnose bipolar, but types was not specified | Research Article |
| Han | 2018 | China | Purpose was to diagnose bipolar, but types was not specified | Research Article |
| Perez | 2018 | UK | Purpose was to diagnose bipolar, but types was not specified | Research Article |
| Osuch | 2018 | China | Purpose was to diagnose bipolar, but types was not specified | Research Article |
| Deng | 2018 | China | Diagnosis of the BD type I, BD Type II | Research Article |
| Jo | 2018 | USA | Purpose was to diagnose bipolar, but types was not specified | Research Article |
| Chung | 2018 | China | Purpose was to diagnose bipolar, but types was not specified | Research Article |
| Liu | 2018 | USA | For BD type II diagnosis | Research Article |
| Chuang | 2017 | China | Purpose was to diagnose bipolar, but types was not mentioned | Research Article |
| Frangou | 2017 | UK | BD type I diagnosis | Research Article |
| Sundaram | 2017 | USA | Purpose was to diagnose bipolar, but types was not specified | Research Article |
| Erguzel | 2016 | Turkey | Purpose was to diagnose bipolar, but types was not mentioned | Research Article |
| Mwangi | 2016 | USA | BD type I, BD Type II Diagnosis | Research Article |
| El Gohary | 2016 | Not available | Purpose was to diagnose bipolar, but types was not specified | Conference Proceedings |
| Saylan | 2016 | Not available | Purpose was to diagnose bipolar, but types was not specified | Conference Proceedings |
| Wu | 2016 | USA | Diagnosis of Euthymic subjects with BD types I or II | Research Article |
