# Supplementary material for: The Role of Machine Learning in Diagnosing Bipolar Disorder: Scoping Review
Source: J Med Internet Res. 2021 Nov 19;23(11):e29749. doi: 10.2196/29749 (PMC8663682; doi:10.2196/29749)
Supplement: Multimedia Appendix 4 [file jmir_v23i11e29749_app4.docx]

**Appendix 4**

Summary of all the data extracted from included studies

| **First author, year** | **Machine learning models** | **Study**  **Settings** | **Bipolar type** | **Data Types** | **Accuracy** | **Other statistics for Validation** | **Data Availability** |
| --- | --- | --- | --- | --- | --- | --- | --- |
| Poletti et al. (2021) | Not mentioned | Existing | Not specified | immune-inflammatory signature | 86.0% | AUC = 97% | Private |
| Idemoto et al. (2021) | Not Mentioned | Existing | Not specified | Blood samples (Serum) | Not mentioned | sensitivity of 51.6% and the specificity of 65.3% | Private |
| Suen et al. (2021) | Elastic Net algorithm | Existing | BD type I, BD Type II & BD not specified | EMR (clinical and demographic variables) | 78.0% | AUC 84%  sensitivity 75%  specificity 81% | Private |
| Sawalha et al. (2021) | SVM | Existing | chronic BD and first-episode BD | Neuropsychological Test | 77.0% | sensitivity 76%  specificity is 77% | Private |
| Parker et al. (2021) | Ensemble Model | Existing | bipolar I, bipolar II | Survey assessing manic/hypomanic symptoms | 91.8% | sensitivity 90.9%  specificity of 96.6% | Private |
| Linke et al. (2020) | Gaussian process classifiers | Existing | Not specified | fractional anisotropy (FA), axial diffusivity (AD), and radial diffusivity (RD) | 75.0% | sensitivity 66.67%  specificity  of 84.21% | Private |
| Sonkurt et al. (2021) | Not mentioned | Novel | Bipolar I | neurocognitive tests | 78.0% | AUC 78%  sensitivity 80 %  specificity 76.2 | Private |
| Mwangi et al. (2016) | RVM | Existing | BD type I, BD Type II | MRI | 70.3% | 66.4% sensitivity  74.2% specificity | Private |
| Cho et al. (2019) | Random forest to develop mood prediction algorithm | Existing | BD type I, BD Type II | Passive digital phenotypes (Heart ryhthms) | 64% and 65% | AUC 67%, and 67% | Private |
| Ma et al. (2019) | Random forest algorithm, support vector regression (SVR), and logistic regression | Existing | Not specified | bipolarity index (BPx) and Affective Disorder Evaluation scale (ADE) | 96.0% | AUC 92.1% | Private |
| Li et al. (2020) | SVM | Existing | Not specified | MRI | 87.5% | sensitivity 86.4%  specificity 88.9% | Private |
| Han et al. (2018) | Not mentioned | existing | Not specified | Not mentioned | Not mentioned | Not mentioned | Not mentioned |
| Jakobsen et al. (2020) | Random Forrest, Deep Neural Network and Convolutional Neural Network algorithms | Existing | Not specified | activity monitoring | 84.0% | 82% sensitivity  84% specificity | Public |
| Laksshman et al. (2017) | DeepBipolar | Novel | Not specified | Genomic data | Not mentioned | AUC 65% | public |
| Chandran et al. (2019) | NLP algorithm | existing | Not specified | EHR (searching key words like YBOCS, OCD, etc.) | Not mentioned | Not mentioned | Public |
| Perez et al. (2018) | Not mentioned | Existing | Not specified | daily mood ratings Survey | 75.0% | Not mentioned | private |
| Achalia et al. (2020) | SVM | Existing | BD type I | MRI, rs-fMRI, diffusion tensor images (neuropsychological measures) | 87.6% | sensitivity of 82.3 %  specificity of 92.7 % | Not mentioned |
| Schwarz et al. (2019) | random forest machine learning and SVM | existing | Not specified | structural and functional MRI | 76.0% | AUC 74% | private |
| Gong et al. (2019) | Neural networks | existing | BD type II | rs-fMRI | Not mentioned | Not mentioned | private |
| Osuch et al. (2018) | SVM | Existing | Not specified | fMRI | 92.4% | sensitivity 87.5%  specificity 97.1% | Private |
| Palaniyappan et al. (2019) | MVAR | existing | psychotic bipolar disorder | rs-fMRI | 96.2% |  | Private |
| Shafquat et al. (2020) | Not mentioned | N/A | Not specified | phenotypes (GWAS datasets) | Not mentioned | AUC 69.7% | Public |
| Frangou et al. (2017) | Support vector machines (SVM) and Gaussian Process Classifier (GPC) | Existing | BD type I | fMRI | 83.5% | sensitivity of 84.6%  specificity of 92.3% | Public |
| Deng et al. (2018) | SVM | Existing | BD type I, BD Type II | MRI- Diffusion tensor images (DTI) | 68.3% | Not mentioned | Private |
| Wu et al. (2016) | Not mentioned | Existing | Euthymic subjects with BD types I or II | CANTAB cognitive scores- measure cognitive performance | 71.0% | Not mentioned | Private |
| Mothi et al. (2019) | Unsupervised machine learning- clustering | Existing | Not specified | MRI | Not mentioned | Not mentioned | Public |
| Jo et al. (2018) | Not mentioned | Existing | Not mentioned | PGBI-10M manic symptom data | Not mentioned | 83% sensitivity  89% specificity | Private |
| Chung et al. (2018) | Baseline logistic regression and RNN classifier | Existing | Not mentioned | Images of faces | Not mentioned | AUC 87.8% | Private |
| El Gohary et al. (2016) | SVM | Existing | Not specified | EEG data | 98.0% | Not mentioned | Not mentioned |
| Saylan et al. (2016) | 1) K Nearest Neighbour Classification Algorithm (K-NN). 2) Decision Tree Algorithm. 3) Naive Bayes algorithm | Existing | Not specified | Microarray expression data set | 86.0% | Not mentioned | Public |
| Liu et al. (2018) | Logistic regression model | Existing | BD type II | sMRI and fMRI | 83%- 89% | AUC 95% | Public |
| Chuang et al. (2017) | random forest (RF) | Existing | Not mentioned | large-scale genome-wide association (GWA) data | 85.2% | Sensitivity 77.7%  Specificity 85.4% | Public |
| Erguzel et al. (2016) | Artificial neural networks - particle swarm optimization (ANN–PSO) | Existing | Not mentioned | Electroencephalography (EEG) | 89.89 % | Sensitivity 83.87 % | Private |

**Abbreviations:** **SVM:** Support Vector Machine**; RF:** Random Forest**; LR:** Logistic Regression**; ANN-PSO:** Artificial neural networks-particle swarm optimization**; GPC:** Gaussian Process Classifier **; FA:** Fractional anisotropy**; AD:** Axial diffusivity**; RD:** Radial diffusivity **; EEG:** Electroencephalography**; GWA:** Large-scale genome-wide association data**; DTI:** Diffusion tensor images**; EHR:** Electronic Health record**; NLP:** Natural Language processing**; MRI:** Magnetic resonance imaging**; fMRI:** Functional magnetic resonance imaging**;**

**rs-fMRI:** Resting state functional magnetic resonance imaging**; CANTAB:** Cambridge neurophysiological test automated battery**; OCD:** Obsessive compulsive disorder**; YBOCS:** Yellow-Brown Obsessive-Compulsive Disorder
