# Supplementary figures and images for: The Role of Machine Learning in Diagnosing Bipolar Disorder: Scoping Review
Source: J Med Internet Res. 2021 Nov 19;23(11):e29749. doi: 10.2196/29749 (PMC8663682; doi:10.2196/29749)

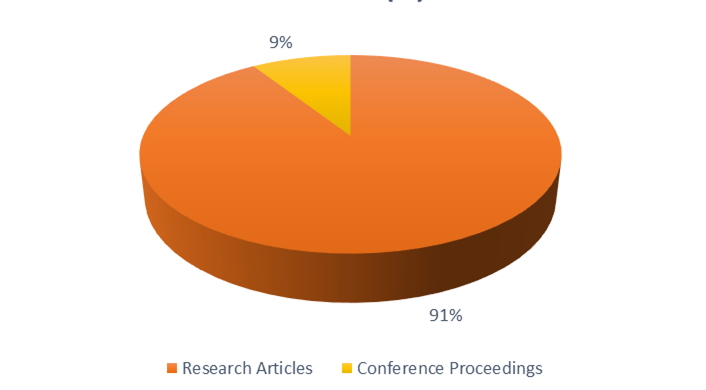

Supplement: Multimedia Appendix 5 [file jmir_v23i11e29749_app5.png]

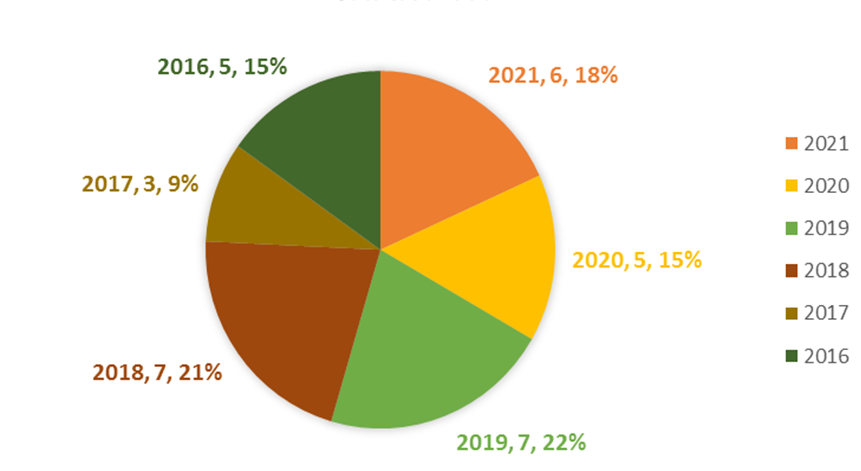

Supplement: Multimedia Appendix 6 [file jmir_v23i11e29749_app6.png]
